# An Explosive Powder

**Published:** 1893-05

**Authors:** 


### An Explosive Powder.

Chlorate of Potash and Tannin are favorite drugs with many dental practitioners. We may therefore draw attention to a letter written to the *Chemist and Druggist*, in which the correspondent warned others of the danger which he himself encountered. A dentist ordered two drachms of chlorate of potash and one drachm of tannin, and when these were mixed together in a mortar there was an explosion.

